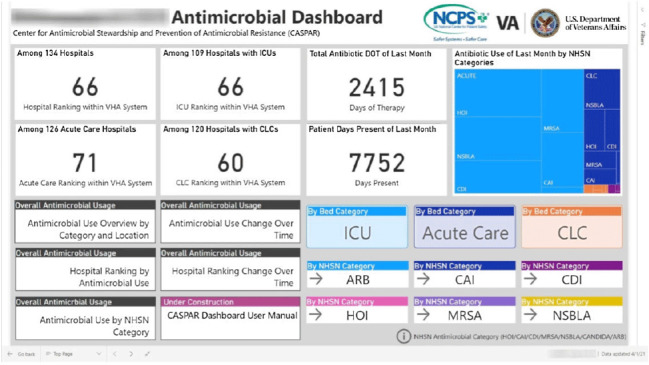# Qualitative Evaluation of an automated nationwide benchmarking antimicrobial utilization dashboard for the VHA

**DOI:** 10.1017/ash.2022.61

**Published:** 2022-05-16

**Authors:** DeShauna Jones, Alexandre Marra, Daniel Livorsi, Eli Perencevich, Michihiko Goto

## Abstract

**Background:** Antimicrobial stewardship programs (ASPs) are advised to audit antimicrobial consumption as a metric to feedback to clinicians. However, many ASPs lack the tools necessary for appropriate risk adjustment and standardized data collection, which are critical for peer-program benchmarking. We evaluated the impact of the dashboard deployment that displays these metrics and its acceptance among ASP members and antimicrobial prescribers. **Materials/methods:** We conducted semistructured interviews of ASP stewards and antimicrobial prescribers before and after implementation of a web-based ASP information dashboard (Fig. 1) implemented in the VA Midwest Health Care Network (VISN23). The dashboard provides risk-adjusted benchmarking, longitudinal trends, and analysis of antimicrobial usage patterns at each facility. Risk-adjusted benchmarking was based on an observed-to-expected comparison of antimicrobial days of therapy at each facility, after adjusting for differences in patient case mix and facility-level variables. Respondents were asked to evaluate several aspects of the dashboard, including its ease of use, applicability to ongoing ASP activities, perceived validity and reliability, and advantages compared to other ASP monitoring systems. All interviews were digitally recorded and transcribed verbatim. The analysis was conducted using MaxQDA 2020.4 and the Consolidated Framework for Implementation Research (CFIR) constructs. **Results:** We completed 4 preimplementation interviews and 11 postimplementation interviews with ASP champions and antimicrobial prescribers from 6 medical centers. We derived 4 key themes from the data that map onto CFIR constructs. These themes were interconnected so that implementation of the dashboard (ie, adapting and adopting) was influenced by respondents’ perception of a facility’s size, patient population, and priority placed on stewardship (ie, structural and cultural context), the availability of dedicated stewardship staff and training needed to implement the dashboard (ie, resources needed), and how the dashboard compared to established stewardship activities (ie, relative advantage). ASP champions and antimicrobial prescribers indicated that dashboard metrics were useful for identifying antimicrobial usage and for comparing metrics among similar facilities. Respondents also specified barriers to acceptance of the risk-adjusted metric, such as disagreement regarding how antimicrobials were grouped by the current NHSN protocol, uncertainty of factors involved in risk adjustments, and difficulty developing a clear interpretation of hospital rankings. **Conclusions:** Given the limited resources for antimicrobial stewardship personnel, automated, risk-adjusted, antimicrobial-use dashboards provided by ASPs are an attractive method to both facilitate compliance and improve efficiency. To increase the uptake of surveillance systems in antimicrobial stewardship, our study highlights the need for clear descriptions of methods and metrics.

**Funding:** None

**Disclosures:** None

**Figure:**